# Rheumatism of the Skin

**Published:** 1842-01

**Authors:** 


*Rheumatism of the Skin.*—The *Archives Generales*, for September, just received, contains a paper on Rheumatism of the Skin, by J. H. S. Bearr, of which we shall present a short summary. After some preliminary remarks on neuralgia of the skin, called, by M. Provoy, dermalgia, he remarks that, in some cases, this neuralgia is evidently rheumatismal, occurring in rheumatic patients, and partaking of other of the characteristics of rheumatic pain. Rheumatism of the skin occurs oftener in men than in women—usually in adults. It is produced by any of the causes of Rheumatism, particularly by exposure to cold. It may be seated in any part of the skin, but is most common on the scalp and upon the lower extremities. The pain is of two kinds—one fixed, the other intermittent; the former is usually slight, the latter more severe. The fixed pain is much augmented by touching the skin even very slightly, and in this way, too, the flying pains, as they are called, may be produced. The skin is not changed in appearance, be the pain ever so severe; sometimes it is dry, sometimes very moist. The duration of the complaint is from two to fourteeen days; the termination, like the accession, is gradual. It frequently returns and varies its point of attack, two distant parts of the skin are, however, very rarely affected at the same time. Rheumatism of the skin often alternates with that of the muscles, the ligaments, or the nerves, but it does not often complicate them. M. B. has known fever to accompany this disease in three cases, which he gives. We shall translate one, abridging it considerably.

*Case.*—A young law student was exposed to a cold rain, and remained in wet clothes for some time. Next morning I found him with fever, thirst, loss of appetite, headache and continued chills; the skin was dry and painful through its whole extent, particularly over the chest and extremities, the pain was lancinating, and was augmented by the slightest touch—even by the chafing of the bed clothes. This pain might have been attributed to rheumatism of the superficial muscles, but the motion of the limb was not painful, except when the skin was brought in contact with something.

The patient was put on the use of demulcents, and the symptoms disappeared in five days.

Rheumatism of the skin may be confounded with that of the muscles, the nerves, or the fibrous tissue of the joints; the diagnostic symptoms are that the pain is superficial, that it intermits, that it is augmented by the slightest pressure, even brushing the skin with the feather of a quill, or touching the hairs by which the affected part may be covered, and that it is not aggravated by motion of the limb. The prognosis is favorable and the treatment the same as that of other rheumatic inflammations.—*N. Y. Med. Gaz.*

